# The changing face of HIV resistance; HIV drug resistance north and south — what's next?

**DOI:** 10.1186/1758-2652-13-S4-O1

**Published:** 2010-11-08

**Authors:** J Schapiro

**Affiliations:** 1 National Haemophilia Center – Israel, Sheba Medical Center, Tel Hashomer, Israel

## 

HIV drug resistance has proved to one of the greatest challenges to effective and durable viral suppression. Selection of drug resistance mutations and widespread cross-resistance between agents of a class are a main obstacle when administering antiretroviral agents in clinical practice. This has been perhaps the greatest barrier to incorporating HIV care in to routine medical management administered by general practitioners. As opposed to many other disease states where the development of effective therapy allowed for simple widespread use as part of general care, HIV drug resistance has mandated cumbersome, resource consuming and demanding medical practice from both clinicians and even more so patients. Extremely demanding lifelong drug taking behavior by patients, high level clinician knowledge and expertise; and expensive monitoring technologies are all required for durable clinical benefit from antiretroviral therapy due to resistance.

But knowledge and understanding of drug resistance has also brought great improvement in HIV care. Technologies to detect and identify resistance as well as rapid and reasonably accurate interpretation have been developed and refined. A far greater understanding of resistance and its consequences by providers and patients have molded our highly effective modern care. Development of improved drugs including those with unique mechanisms has greatly benefited from our growing knowledge.

As we move forward to the next decade of HIV care, we need to revisit how we relate to and address HIV drug resistance. Old assumptions need to be challenged; data needs to be critically evaluated considering our new and improved drugs, and widespread treatment of patients in resource limited settings need to be specifically prioritized as challenges may not be identical. How should our much improved (but expensive) resistance assays be used? To what degree do we need to continue to closely monitor HIV drug resistance and in what settings? Is resistance still a high priority when designing optimal drug combinations for our patients – those naive and those drug-experienced? How can we minimize the barrier resistance presents to more simplified and widespread antiretroviral therapy? These are important issues we must address to guarantee the best care for the most patients in coming years.

